# Assessing the Efficacy of Inferior Vena Cava Collapsibility Index for Predicting Hypotension after Central Neuraxial Block: A Systematic Review and Meta-Analysis

**DOI:** 10.3390/diagnostics13172819

**Published:** 2023-08-31

**Authors:** Ying-Jen Chang, Chien-Cheng Liu, Yen-Ta Huang, Jheng-Yan Wu, Kuo-Chuan Hung, Ping-Hsin Liu, Chien-Hung Lin, Yao-Tsung Lin, I-Wen Chen, Kuo-Mao Lan

**Affiliations:** 1 Department of Anesthesiology, Chi Mei Medical Center, Tainan City 71004, Taiwan; 2 Department of Recreation and Health-Care Management, College of Recreation and Health Management, Chia Nan University of Pharmacy and Science, Tainan 71710, Taiwan; 3 Department of Anesthesiology, E-Da Hospital, I-Shou University, Kaohsiung City 82445, Taiwan; 4 Department of Nursing, College of Medicine, I-Shou University, Kaohsiung City 82445, Taiwan; 5 School of Medicine, I-Shou University, Kaohsiung City 82445, Taiwan; 6 Department of Surgery, National Cheng Kung University Hospital, College of Medicine, National Cheng Kung University, Tainan City 70101, Taiwan; 7 Department of Nutrition, Chi Mei Medical Center, Tainan City 71004, Taiwan; 8 Department of Anesthesiology, E-Da Dachang Hospital, I-Shou University, Kaohsiung City 80794, Taiwan; 9 Department of Anesthesiology, Chi Mei Medical Center, Liouying, Tainan City 71004, Taiwan

**Keywords:** inferior vena cava collapsibility index, hypotension, spinal anesthesia, meta-analysis, central neuraxial block

## Abstract

The use of ultrasonography to predict spinal-induced hypotension (SIH) has gained significant attention. This diagnostic meta-analysis aimed to investigate the reliability of the inferior vena cava collapsibility index (IVCCI) in predicting SIH in patients undergoing various surgeries. Databases, including Embase, Cochrane Library, Medline, and Google Scholar, were screened until 28 July 2023, yielding 12 studies with 1076 patients (age range: 25.6–79 years) undergoing cesarean section (CS) (*n* = 4) or non-CS surgeries (*n* = 8). Patients with SIH had a significantly higher IVCCI than those without SIH (mean difference: 11.12%, 95% confidence interval (CI): 7.83–14.41). The pooled incidence rate of SIH was 40.5%. IVCCI demonstrated satisfactory overall diagnostic reliability (sensitivity, 77%; specificity, 82%). The pooled area under the curve (AUC) was 0.85, indicating its high capability to differentiate patients at risk of PSH. The Fagan nomogram plot demonstrated a positive likelihood ratio (PLR) of 4 and a negative likelihood ratio (NLR) of 0.28. The results underscore the robustness and discriminative ability of IVCCI as a predictive tool for SIH. Nevertheless, future investigations should focus on assessing its applicability to high-risk patients and exploring the potential enhancement in patient safety through its incorporation into clinical practice.

## 1. Introduction

Spinal anesthesia is a popular and reliable anesthetic approach for cesarean section or lower extremity surgery; however, hypotension after this technique may occur, with an incidence of 8.2% to 64% [1,2]. Spinal-induced hypotension is mainly caused by sympathetic blockade, reduced systemic vascular resistance, and reduced venous return [3]. Although relatively healthy patients can tolerate post-spinal hypotension well, their occurrence may elevate the risk of serious complications, such as myocardial infarction, postoperative delirium, and even mortality [4,5,6,7,8,9]. Although fluid administration and/or vasopressor agents are often used for the prophylaxis of spinal-induced hypotension, these preventive strategies may pose certain risks, such as volume overload or reactive hypertension. Perioperative crystalloid administration, when used in large quantities, can have significant side effects on various organ systems, including impaired left ventricular stroke volume, increased risk of myocardial ischemia, pulmonary function impairment, prolonged paralytic ileus, reduced tissue oxygenation, and elevated risk of electrolyte disturbances [10,11,12,13]. Similarly, the use of fixed-rate norepinephrine or phenylephrine infusions has been associated with the occurrence of reactive hypertension, with reported incidences ranging from 4.8% to 7.3% [14]. The ability to predict the likelihood of spinal-induced hypotension offers significant advantages to anesthesiologists, leading to improved patient outcomes and enhanced postoperative recovery.

The use of ultrasound to predict the development of spinal-induced hypotension has attracted considerable interest recently [15,16,17,18]. This non-invasive and real-time imaging technique offers valuable insights into hemodynamic changes, enabling clinicians to proactively anticipate and prepare for perioperative hypotensive episodes [19]. The inferior vena cava (IVC) collapsibility index was used as a quantitative measure to assess the extent of IVC collapse during inspiration. It is calculated using the following formula: IVC collapsibility index = (maximum dimension on expiration − minimum dimension on inspiration)/maximum dimension on expiration [20]. In spontaneously breathing individuals, the IVC collapsibility index demonstrated acceptable diagnostic reliability (sensitivity, 71%; specificity, 81%) for predicting volume responsiveness [20]. Numerous recent studies have consistently reported that this technique can be used to predict the likelihood of spinal-induced hypotension [21,22,23]. Nevertheless, there has been considerable variation in the diagnostic reliability of the IVC collapsibility index across studies [21,22,23,24,25], highlighting the importance of conducting a systematic evaluation of its efficacy. The purpose of this diagnostic meta-analysis was to examine the diagnostic accuracy of the IVC collapsibility index in anticipating spinal-induced hypotension in patients undergoing various surgeries.

## 2. Materials and Methods

### 2.1. Study Protocol

This diagnostic meta-analysis, adhering to the Preferred Reporting Items for Systematic Reviews and Meta-Analyses of Diagnostic Test Accuracy Studies (PRISMA-DTA) guidelines, was previously registered at PROSPERO.

### 2.2. Data Source and Literature Search

In this investigation, two separate authors conducted a comprehensive exploration of four databases, Cochrane Library, Embase (OVID), Medline (OVID), and Google Scholar, covering the period from their establishment to 28 July 2023. The primary objective of this study was to identify pertinent studies that employed ultrasound-derived parameters, specifically focusing on the IVC collapsibility index, to predict the probability of spinal-induced hypotension in the perioperative period. Language and country restrictions were not applied during the literature search process. Google Scholar was utilized as a supplementary resource in conjunction with these databases.

During our literature search, the following keywords were used: (“Ultrasonography” or “Ultrasound-guided” or “Ultrasound” or “Sonography” or “Echography” or “Echotomography” or “Ultrasonic”) and (“Post-Spinal” or “Subarachnoid block” or “Spinal block” or “Intrathecal anesthesia” “Subarachnoid anesthesia” or “Intrathecal block” or “Spinal-epidural anesthesia” or “neuraxial anesthesia” or “Intraspinal anesthesia”) and (“Vena cava” or “vena cava collapsibility index” or “vena cava inferior diameter” or “venacaval collapsibility index” or “vena cava diameter” or “vena cava collapse index” or “vena cava dimensions”) and (hypotension). In addition, manual selection was performed to identify studies that potentially met the eligibility criteria. In instances of any discrepancies or conflicts that emerged during the screening process between the two authors, a consensus-based approach was employed to reach a resolution with the involvement of a third team member. Appendix A provides additional information pertaining to the search strategy employed for one of these databases (i.e., Medline).

### 2.3. Inclusion and Exclusion Criteria

Studies were considered eligible for inclusion based on the following criteria: (1) adult patients who underwent central neuraxial block, such as spinal anesthesia, irrespective of the surgical procedure, (2) utilization of the IVC collapsibility index as a predictive tool for hypotension, and (3) availability of relevant information on the number of patients experiencing spinal-induced hypotension, sensitivity, and specificity. Eligible study designs included cohort studies, randomized controlled trials, and case–control studies.

Studies were excluded from the analysis if they met any of the following criteria: (1) those published as case reports, abstracts, letters, conference papers, or review papers; (2) studies investigating non-surgical individuals or pediatric patients; (3) studies without relevant outcomes; and (4) studies without accessible full text.

### 2.4. Extraction of Data

Data retrieval from individual studies was conducted independently by two teams, and any disagreement was resolved through discussion. The following details were obtained: study variables, such as number of participants, name of the primary author, demographics of patients (e.g., sex distribution), sensitivity and specificity values, IVC collapsibility index data, number of patients experiencing spinal-induced hypotension, and country of origin. In instances where information was missing, attempts were made to acquire relevant data by reaching out to the corresponding author of the included articles.

### 2.5. Outcomes of Interest and Definitions

The primary purpose of this diagnostic meta-analysis was to explore the diagnostic reliability of the IVC collapsibility index for detecting the probability of spinal-induced hypotension immediately after central neuraxial block. The definitions of spinal-induced hypotension were aligned to those used in each study. In cases where studies reported relevant outcomes in patients in either the supine or left-lateral position, we adopted the data obtained from the supine position for our analysis.

### 2.6. Assessment of Study Quality

The Quality Assessment for Diagnostic Accuracy Studies-2 (QUADAS-2) tool was used to assess the methodological quality of each study [26]. The ‘applicability concerns’ category encompasses three domains, whereas the ‘risk of bias’ category includes four domains. Both team members conducted an independent subjective review of the included studies, assessing each domain with ratings of “low risk”, “unclear”, or “high risk”. Any discrepancies that arose during this assessment process were addressed through extensive discussion until a consensus was reached. In instances where reaching a consensus proved challenging, a third author was engaged in facilitating the resolution of the disagreements.

### 2.7. Statistical Analysis

Continuous variables were expressed as the mean difference (MD) along with a 95% confidence interval (CI), and the Mantel–Haenszel random-effects model was employed, considering the heterogeneity present in the study population. The degree of heterogeneity was evaluated using I^2^ statistics and was considered significant when it exceeded 50% [27]. Data were synthesized using the Cochrane Review Manager (RevMan 5.3; Copenhagen: The Nordic Cochrane Centre). Meta-regression was used to assess the association between patient age and ICV collapsibility index using Comprehensive Meta-Analysis (CMA) V3 software (Biostat, Englewood, NJ, USA).

The diagnostic efficacy of the ICV collapsibility index was assessed by computing the area under the curve (AUC) from a summary receiver operating characteristic (sROC) curve. To estimate post-test probability, Fagan’s nomogram with likelihood ratios (LRs) of the test results was used. A Deek’s funnel plot was used to explore the presence of publication bias. Statistical significance was determined at a threshold of 0.05, and all statistical analyses were conducted using the MIDAS command in Stata 15 (StataCorp LLC., College Station, TX, USA).

## 3. Results

### 3.1. Study Selection and Study Characteristics

**Figure 1:**
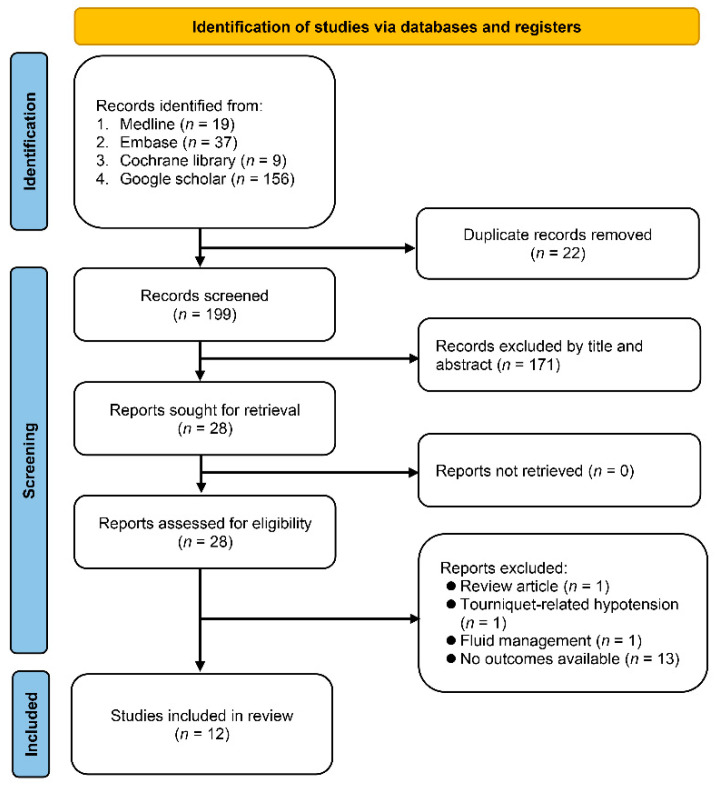
Flow chart for selection of studies in current meta-analysis.

A literature search was conducted across four electronic databases, Medline (OVID), Embase (OVID), Google Scholar, and Cochrane Library, resulting in the retrieval of 221 studies. After excluding duplicate records (22 in total) and screening titles and abstracts to eliminate unsuitable ones (171 in total), a final selection of 28 reports was identified for further analysis. Review articles (*n* = 1), studies focusing on tourniquet-related hypotension (*n* = 1) or fluid assessment (*n* = 1), and those lacking available outcome data (*n* = 13) were subsequently excluded, leaving a final total of 12 studies with 1076 patients for the final review (Figure 1) [18,21,22,23,24,25,28,29,30,31,32,33].

**Figure 2:**
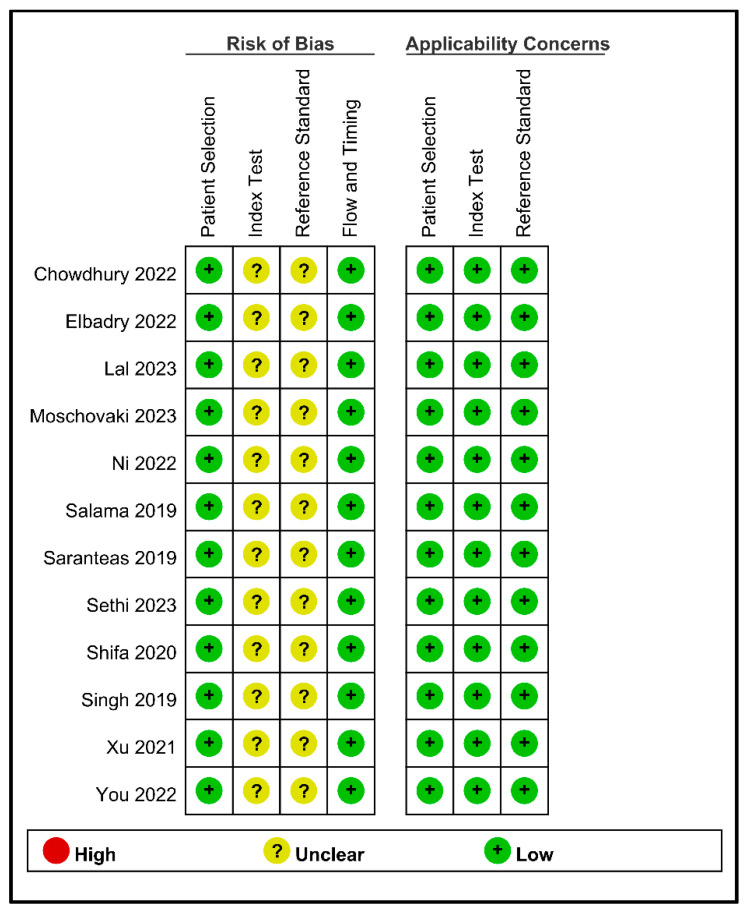
A summary of the methodological quality of the 12 studies [18,21,22,23,24,25,28,29,30,31,32,33].

Table 1 provides a comprehensive overview of the characteristics of the 12 studies. In the 12 studies, the age range of the patients was 25.6 to 79 years. Eight studies [18,22,23,24,28,29,30,33] included male and female populations, with male proportions varying from 45% to 77.3%, while four studies [21,25,31,32] specifically focused on females undergoing cesarean section. Body mass index (BMI) was reported in nine studies and ranged from 23.2 to 29.2 [18,21,22,23,28,29,31,32,33]. Three studies provided data on body weight, with values ranging from 60 to 64.9 kg [24,25,30]. Ten studies focused on patients classified as ASA I–II [18,21,22,23,24,25,30,31,32,33], while two other studies specifically investigated patients with ASA II–III [28,29]. These studies had a wide range of sample sizes, ranging from 40 to 299 patients. The information regarding the dosages of local anesthesia utilized and the corresponding levels of anesthesia achieved across the various studies is summarized in Table 2. While there was variation in the specific type of local anesthetics used and their respective dosages, a consistent trend was observed: the majority of studies aimed to restrict the dermatome level of spinal anesthesia to below T6. Each study used different threshold values ranging from 20.4 to 50. Regarding the performance of the predictive models, three studies showed an area under the curve (AUC) of less than 0.7, with values ranging from 0.38 0.672. In contrast, the other nine studies demonstrated an AUC greater than 0.7, with values ranging from 0.72 0.941. Figure 2 presents an overview of the methodological quality of the 12 studies included in this analysis. All studies were considered to have an unclear risk of bias in the index test domain owing to the absence of a predetermined cutoff value before the study. Similarly, the risk for the reference standard is unclear, as the definition of hypotension varies among studies.

### 3.2. Outcomes

#### 3.2.1. Association of Spinal-Induced Hypotension with Inferior Vena Cava Collapsibility Index

**Figure 3:**
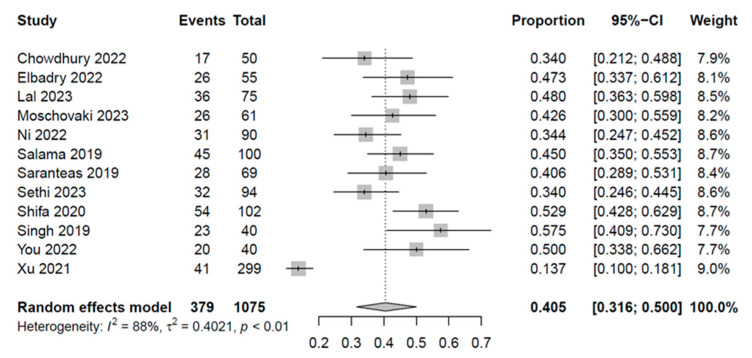
Forest plot illustrating the pooled spinal-induced hypotension incidence. CI: confidence interval [18,21,22,23,24,25,28,29,30,31,32,33].

**Figure 4:**
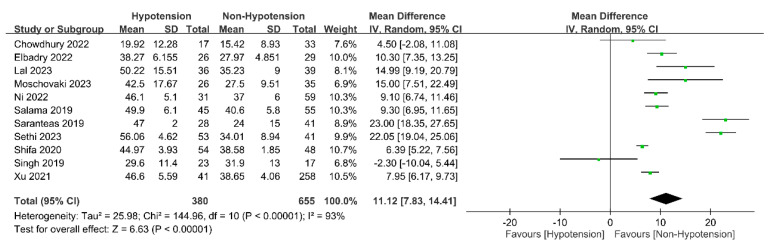
Forest plot showing the mean difference in inferior vena cava (IVC) collapsibility index between patients with or without spinal-induced hypotension. IV: inverse variance; CI: confidence interval [18,21,22,23,24,25,28,29,30,31,33].

**Figure 5:**
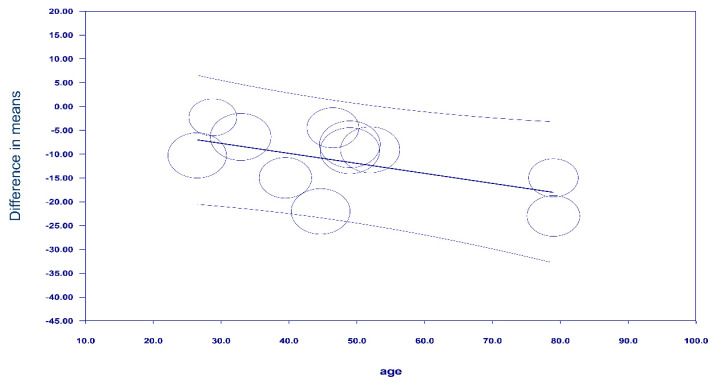
Meta-regression plot showing the association between patient age and inferior vena cava (IVC) collapsibility index [18,21,22,23,24,25,28,29,30,31,33].

Eleven of the twelve studies [18,21,22,23,24,25,28,29,30,31,32] found that approximately 1/3 to 1/2 of their patients suffered from hypotension (range: 34–57.5%), while one report [33] reported a 13.71% incidence rate (Figure 3). Patients with spinal-induced hypotension had a significantly higher IVC collapsibility index than those without spinal-induced hypotension (mean difference: 11.12%, 95% CI: 7.83 to 14.41, *p* < 0.00001, I^2^ = 93%) (Figure 4). Meta-regression analysis showed a correlation between patient age and the IVC collapsibility index (coefficient: −0.21, *p* = 0.0385) (Figure 5).

#### 3.2.2. Diagnostic Efficacy of Inferior Vena Cava (IVC) Collapsibility Index for Predicting Spinal-Induced Hypotension

**Figure 6:**
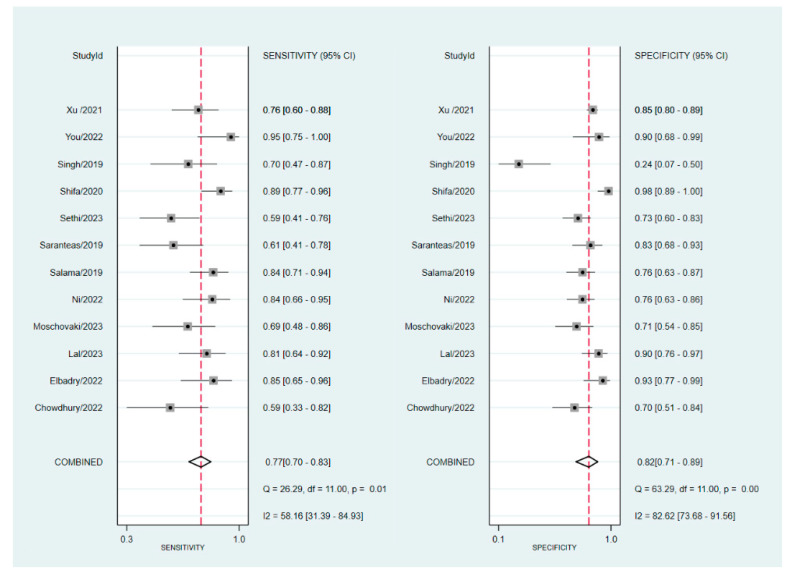
The forest plot displays the diagnostic efficacy of the inferior vena cava (IVC) collapsibility index as a predictive measure for spinal-induced hypotension [18,21,22,23,24,25,28,29,30,31,32,33].

**Figure 7:**
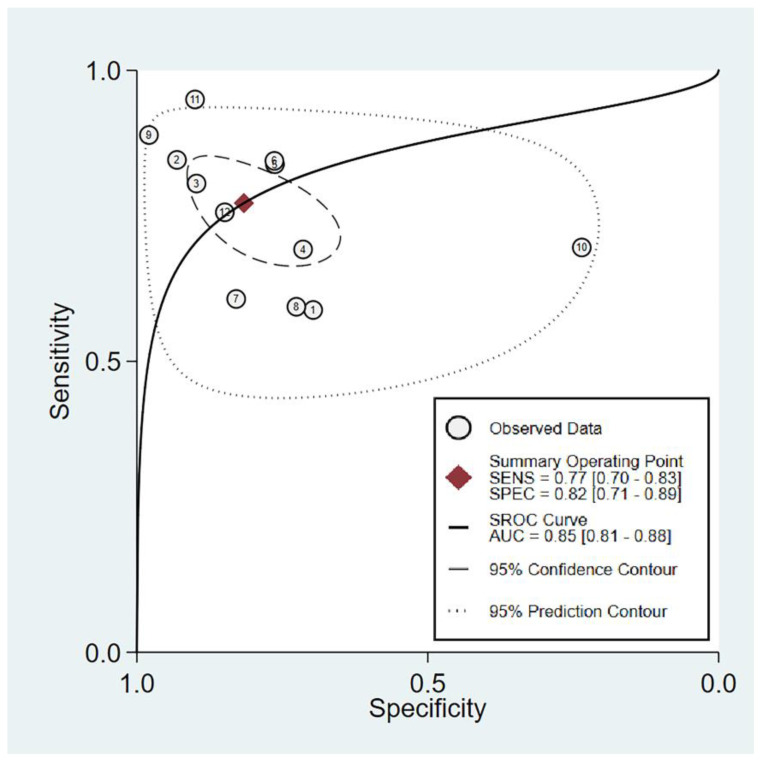
The effectiveness of the inferior vena cava (IVC) collapsibility index for prediction of spinal-induced hypotension is investigated using summary receiver operating characteristic (sROC) curve analysis. The unfilled circles on the graph indicate the individual study estimates of sensitivity and (1-specificity). The solid line on the graph represents the weighted sROC curve, while the diamond symbolizes the pooled outcome. SENS and SPEC represent sensitivity and specificity, respectively [18,21,22,23,24,25,28,29,30,31,32,33].

**Figure 8:**
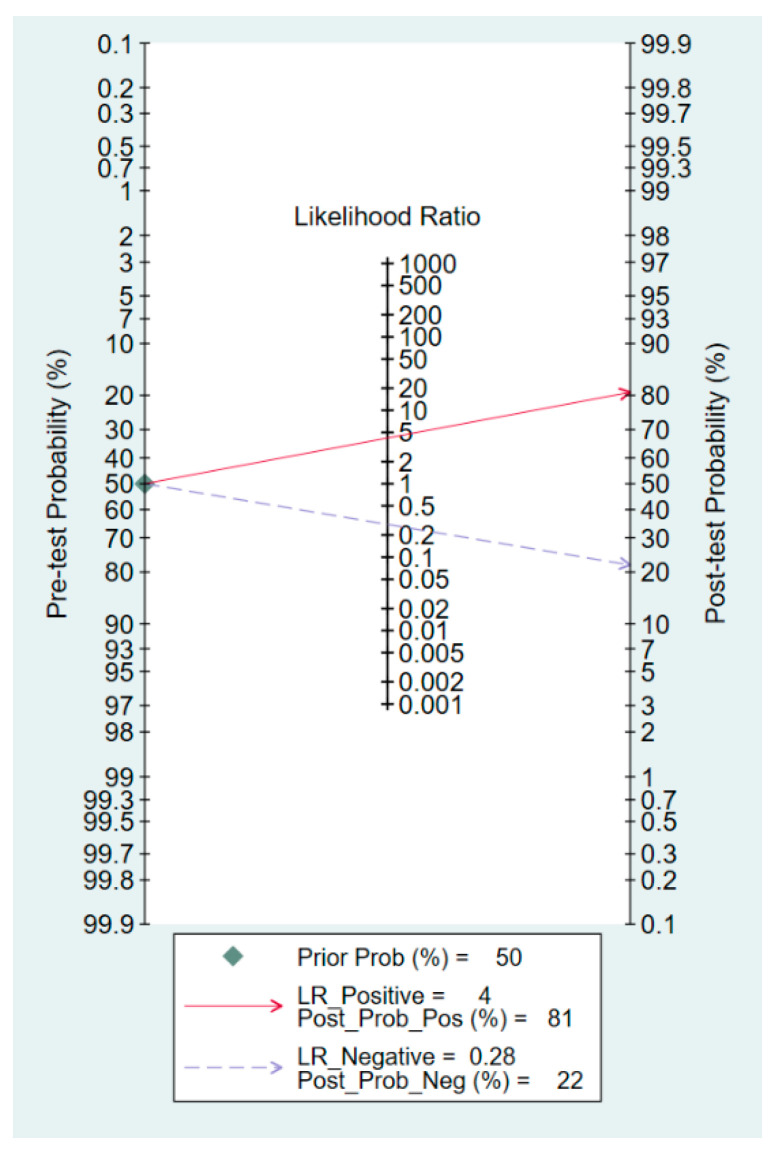
Fagan’s nomogram plot illustrates the usefulness of the inferior vena cava (IVC) collapsibility index to predict spinal-induced hypotension. Pos, positive; Neg, negative; LR, likelihood ratio; Prob, probability [18,21,22,23,24,25,28,29,30,31,32,33].

**Figure 9:**
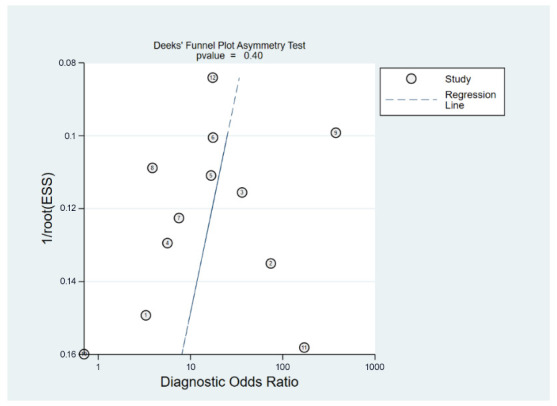
The presence of publication bias among the studies in the analysis was evaluated using Deek’s funnel plot asymmetry test, suggesting a low risk of publication bias (*p* = 0.4) [18,21,22,23,24,25,28,29,30,31,32,33].

The sensitivity and specificity of the individual studies are shown in Figure 6. In five studies [24,25,28,29,30], the sensitivity values were below 70%, ranging from 58.8% to 69%. In contrast, in the remaining seven studies [18,21,22,23,31,32,33], the sensitivity values were above 70%, with a range of 75.6% to 95%. The specificity values across studies also showed considerable variation, ranging from 23.5% to 98.8%. The overall sensitivity was 77% (95% confidence interval: 70–83%), while the overall specificity was 82% (95% confidence interval:71–89%) (Figure 6) [18,21,22,23,24,25,28,29,30,31,32,33]. The pooled AUC was 0.85, suggesting a high probability of distinguishing between subjects with spinal-induced hypotension and those without (Figure 7). The predictive capacity of the IVC collapsibility index for hypotension was assessed using Fagan nomograms. The positive and negative likelihood ratios were found to be 4 and 0.28, respectively (Figure 8). Given an initial pretest probability of 50%, the incorporation of the IVC collapsibility index as a diagnostic test yielded post-test probabilities of 81% for a positive result and 22% for a negative result (Figure 8). Additionally, Deek’s funnel plot asymmetry test revealed a low risk of publication bias regarding the diagnostic efficacy of the IVC collapsibility index (*p* = 0.4) (Figure 9) [18,21,22,23,24,25,28,29,30,31,32,33].

## 4. Discussion

This non-invasive ultrasound-guided technique has shown promise in assessing cardiac preload and intravascular volume status [20,34], offering potential benefits for the early detection and management of hypotensive episodes during spinal anesthesia. The analysis of 12 studies with a total of 1076 patients revealed promising results for the IVC collapsibility index in predicting spinal-induced hypotension. The overall sensitivity and specificity were 77% and 82%, respectively. The pooled area under the curve (AUC) was 0.85, suggesting that the IVC collapsibility index has a high probability of effectively distinguishing between patients who are likely to experience hypotension and those who are not. This finding underscores the robustness and discriminative ability of the IVC collapsibility index as a predictive tool for spinal-induced hypotension across the analyzed studies. This predictive ability can help clinicians monitor and manage patients who may be susceptible to hypotensive episodes during spinal anesthesia.

Intraoperative hypotension, irrespective of its etiology, may have detrimental effects on patients’ well-being in both immediate and prolonged periods. A sudden drop in blood pressure reduces perfusion to vital organs, making patients prone to arrhythmia, myocardial ischemia or infarction, acute kidney injury, and postoperative delirium [4,6,35,36,37,38,39]. In a recent study of 216 women undergoing cesarean section, hypotension was observed in 79.6% of the cases, and a statistically significant association was found between hypotension and arrhythmia [35]. A retrospective cohort study involving 5127 patients undergoing noncardiac surgery found an association between postoperative acute kidney injury and prolonged intraoperative periods of mean arterial pressure (MAP) below 55 and 60 mmHg [39]. A recent report of 605 elderly individuals who underwent orthopedic and thoracic surgery revealed an incidence of postoperative delirium in 14.7% of cases occurring within three days postoperatively, and the duration of hypotension exhibited a non-linear and “inverted L-shaped” relationship with the development of postoperative delirium [5]. Consistently, in an analysis of 316,717 patients undergoing noncardiac surgery, a mean arterial pressure (MAP) < 55 mmHg was linked to a progressively higher likelihood of experiencing postoperative delirium [6]. These findings underscore the immediate detrimental effect of untreated hypotension on patient health. In a cohort of 135 patients without pre-existing dementia, a previous study demonstrated that postoperative delirium served as an independent predictor of new-onset dementia within a 3-year post-surgery period [37]. This finding further suggests that the adverse consequences of untreated hypotension might manifest indirectly through other complications such as postoperative delirium, contributing to the subsequent development of dementia in susceptible individuals.

In the current meta-analysis, with an overall sensitivity of 77% and specificity of 82%, the IVC collapsibility index demonstrated the potential to predict the occurrence of hypotension. Several studies have used ultrasound-guided assessment of the internal jugular vein (IJV) or the carotid artery to predict spinal-induced hypotension. A previous study of 47 patients undergoing elective surgery with spinal anesthesia revealed that the IJV collapsibility index had unsatisfactory efficacy (sensitivity, 64%; specificity, 63.6%) in predicting spinal-induced hypotension [40]. In another study, on patients aged > 65 years scheduled for elective surgery under spinal anesthesia, ultrasonographic Carotid Artery Flow Measurements demonstrated improved diagnostic reliability (sensitivity: 76.5%; specificity: 75.8%) [16]. Compared to these techniques, the IVC collapsibility index may be favorable in clinical practice. Furthermore, a study reported that this technique is easy to perform [41], indicating its feasibility in clinical practice. The strength of our meta-analysis lies in its inclusion of a diverse patient population, including those undergoing both cesarean section and non-cesarean section procedures, supporting its generalizability to a wider range of patients.

Given that the IVC collapsibility index was assessed prior to spinal anesthesia ad-ministration across all studies, it reflects the patients’ baseline hydration status rather than a direct effect of the spinal anesthesia. Nevertheless, the occurrence of hypotension subsequent to spinal anesthesia is probably influenced by a combination of factors, including the sympathetic blockade induced by the procedure and the patient’s volume status. Spinal anesthesia can lead to a decrease in sympathetic tone, resulting in vasodilation and the potential for hypotension, even in patients who were not hypovolemic before the procedure. Consequently, relying solely on the baseline IVC collapsibility index may not entirely capture the actual risk of hypotension following spinal anesthesia, as factors associated with the procedure itself can impact blood pressure fluctuations. Additional research is warranted to establish the optimal approach for integrating data from the IVC collapsibility index with other potential risk factors for hypotension arising from spinal anesthesia.

As the occurrence of hypotension following spinal anesthesia is likely linked to the sympathetic blockade induced by the procedure, it is plausible that the type and dosage of local anesthetic drugs could play a role. Nevertheless, it is important to note that the majority of the studies included in our meta-analysis purposefully aimed to minimize potential effects related to anesthetic drug administration by limiting the extent of anesthesia to levels below the T6 dermatome (Table 2). While variations in anesthetic protocols do exist, any potential influence stemming from anesthetic drug administration is unlikely to significantly distort the findings. In addition to the potential influence of anesthetic drugs, it is important to consider the impact of patients’ comorbidities on our outcomes. For instance, individuals with a history of neurological diseases might be more susceptible to hypotensive episodes following spinal anesthesia. Upon a comprehensive review of the included studies, while none explicitly detailed the inclusion of patients with neurological events in their populations, a noteworthy observation emerged. The majority of the selected studies primarily focused on enrolling patients with relatively favorable health profiles, often classified as ASA I–II (Table 1). Furthermore, our examination of the studies revealed a consistent effort to exclude individuals with a history of hemodynamic instability, hypertension, or cardiovascular disease (Supplemental Table S1). Therefore, most studies attempted to minimize the impact of pre-existing conditions that could confound the assessment of hypotension-related factors.

In the current meta-analysis, 10 out of 12 studies focused on relatively healthy patients with ASA I-II; however, approximately 1/3 to 1/2 of the patients in these studies experienced hypotension, highlighting the common occurrence of intraoperative hypotension, even in relatively healthy patients. Several risk factors for hypotension after spinal anesthesia have been identified, including chronic alcohol consumption, a history of hypertension, body mass index, sensory block height, and urgency of surgery [1]. In addition, a previous meta-analysis of 3086 patients undergoing elective cesarean section reported a hypotension incidence of up to 80% [42]. Considering the high incidence of hypotension in patients undergoing cesarean section and those at high risk, the use of the IVC collapsibility index appears to be a valuable approach for managing and mitigating the risks associated with intraoperative hypotension.

The prediction of spinal-induced hypotension occurrence can be facilitated by employing various monitors, which may include the analysis of arterial waveforms, heart rate variability (HRV), and pupillometry [43,44,45,46]. The Hypotension prediction Index (HPI), which employs the arterial waveform to predict the occurrence of intraoperative hypotension, demonstrated a predictive capability for hypotensive events in women undergoing cesarean delivery, with a sensitivity of 83% and specificity of 83% when assessed three minutes before the occurrence of the event [43]. Preoperative HRV analysis in individuals undergoing elective cesarean delivery under spinal anesthesia showed that a high LF/HF ratio before spinal anesthesia predicted severe hypotension, indicating its potential for identifying at-risk patients [44]. In a prior investigation, the effectiveness of pupillometry as a prognostic indicator for hypotension after spinal anesthesia during caesarean section was examined in a cohort of 141 patients (70%) [46]. The study findings revealed that the sensitivity and specificity of pupillometry in predicting hypotension were 60% and 70%, respectively [46]. The potential drawbacks of these techniques as predictive methods for hypotension in the operating room include limited availability and concerns regarding their accuracy.

This meta-analysis has some limitations that may impact the generalizability and clinical application of the diagnostic approach for predicting spinal-induced hypotension. First, the majority of patients studied were not obese, making it uncertain how well the test is performed in this specific population, where obesity is a known risk factor for hypotension [1]. Second, the inclusion of only two studies with ASA II-III patients limits the assessment of the efficacy of the test in ill individuals, who may have additional factors contributing to hypotension. Third, the wide variation in sample sizes across studies, ranging from 40 to 299, may have introduced bias to the overall results. Finally, the use of different cutoff values for the test in various studies complicates its application in clinical practice. To address these limitations and improve the utility of the test, further research is necessary to investigate optimal cutoff values and include diverse patient populations representative of real-world scenarios.

## 5. Conclusions

The analysis of 12 studies comprising 1076 patients yielded promising results regarding the predictive capacity of the IVC collapsibility index for spinal-induced hypotension. The sensitivity and specificity were 77% and 82%, respectively, indicating a favorable ability of the IVC collapsibility index to differentiate between patients at risk of experiencing hypotension and those who are not. Nevertheless, future investigations should focus on assessing its applicability to high-risk patients and exploring the potential enhancement of patient safety through its incorporation into clinical practice.

## Figures and Tables

**Table 1:** Characteristics of studies (*n* = 12).

Studies	Age (Years)	Male (%)	BMI (kg/m^2^) or Weight (kg)	ASA	*n*	Surgery	Cut-Off Value ⁋	HI	AUC	Country
Chowdhury 2022 [24]	46.5	76	60	I–II	50	Non-CS	21.25	34%	0.408	India
Elbadry 2022 [21]	26.5 vs. 25.6 †	0	29 vs. 29 †	I–II	55	CS	33	47.3%	0.891	Egypt
Lal 2023 [22]	39.5	77.3	23.29	I–II	75	Non-CS	43.5	48%	0.828	India
Moschovaki 2023 [28]	79 vs. 78.5 †	45.2	31 vs. 30 †	II–III	61	Non-CS	28	42.6%	0.72	Greece
Ni 2022 [23]	52	53.3	23.2	I–II	90	Non-CS	42	34.4%	0.834	China
Salama 2019 [18]	49	45	24.5	I–II	100	Non-CS	44.7	45%	0.84	Egypt
Saranteas 2019 [29]	79	44.3	29.2	II–III	70	Non-CS	30	40.6%	0.77	Greece
Sethi 2023 [30]	44.7 vs. 36.8 †	61.7	62 vs. 62 †	I–II	94	Non-CS	50	34%	0.672	India
Shifa 2020 [31]	32.9	0	27.6	I–II	102	CS	41.43	52.9%	0.941	China
Singh 2019 [25]	28.8	0	64.9	I–II	40	CS	20.40	57.5%	0.38	India
You 2022 [32] ‡	30	0	27.0	I–II	40	CS	21.69	50%	0.93	China
Xu 2021 [33]	49 vs. 47 †	59.9	24 vs. 24 †	I–II	299	Non-CS	43.26	13.71%	0.864	China

‡ Combine spinal-epidural; HI: hypotension incidence; CS: Caesarean Section; ASA: American Society of Anesthesiologists; † presented as hypotension vs. non-hypotension; AUC: area under curve; ⁋ cut-off value of inferior vena cava collapsibility index in predicting post-spinal hypotension.

**Table 2:** Dosage of local anesthetics and level of spinal anesthesia.

	Dosage of Local Anesthetics	Level of Spinal Anesthesia
Chowdhury 2022 [24]	Bupivacaine 0.5%, 12.5 mg	T6–T8 dermatomes
Elbadry 2022 [21]	Bupivacaine 0.5%, 12.5 mg	Reached T6
Lal 2023 [22]	Bupivacaine 0.5%, 14 mg	Reached T6
Moschovaki 2023 [28]	Ropivacaine 0.75%, 12–18 mg	Reached T6–T7
Ni 2022 [23]	Bupivacaine 0.5%, 12–15 mg	Reached T6–T8
Salama 2019 [18]	Bupivacaine 0.5%, 12–15 mg	T8–T10
Saranteas 2019 [29]	Ropivacaine 0.75%, 12–18 mg	NA
Sethi 2023 [30]	Bupivacaine 0.5%, 10–12.5 mg	T10
Shifa 2020 [31]	NA	NA
Singh 2019 [25]	Bupivacaine 0.5%, 8 mg	Reached T6 level
You 2022 [32]	Ropivacaine 0.5%, 12.5 mg	Range of T4–T6
Xu 2021 [33]	Naropin 1%, 15 mg	NA

NA: not available.

## Data Availability

The original contributions presented in this study are included in this article; further inquiries can be directed to the corresponding authors.

## Supplementary Materials

The following supporting information can be downloaded at: https://www.mdpi.com/article/10.3390/diagnostics13172819/s1, Table S1: Exclusion criteria for participants in individual study.

## Appendix A. Search Strategy for Medline

1	(“Ultrasound” or “Ultrasound-guided” or “Ultrasonography” or “Echography” or “Sonography” “Ultrasonic” or “Echotomography”).mp.
2	exp “Ultrasonography”/
3	(“Post-Spinal” or “Subarachnoid block” or “Spinal block” or “Subarachnoid anesthesia” or “Intrathecal block” or “Intrathecal anesthesia” or “Spinal-epidural anesthesia” or “Intraspinal anesthesia” or “neuraxial anesthesia”).mp.
4	exp “Anesthesia, Spinal”/
5	(“Vena cava” or “vena cava collapsibility index” or “vena cava inferior diameter” or “venacaval collapsibility index” or “vena cava diameter” or “vena cava collapse index” or “vena cava dimensions”).mp.
6	exp “Vena Cava, Inferior”/
7	(“Hypotension” or “Low blood pressure”).mp.
8	exp “Hypotension”/
9	(1 or 2) and (3 or 4) and (5 or 6) and (7 or 8)
