# Supplementary material for: Assessing the Efficacy of Inferior Vena Cava Collapsibility Index for Predicting Hypotension after Central Neuraxial Block: A Systematic Review and Meta-Analysis
Source: Diagnostics (Basel). 2023 Aug 31;13(17):2819. doi: 10.3390/diagnostics13172819 (PMC10487093; doi:10.3390/diagnostics13172819)
Supplement: Supplementary file 1 [file diagnostics-13-02819-s001.zip › diagnostics-2570772-supplementary.pdf]

Supplemental Table S1. Exclusion criteria for participants in individual study

Chowdhury 2022 [24]	We excluded the patients who were hypertensive or had any cardiac disease
Elbadry 2022 [21]	Cases with emergency conditions and uncontrolled systemic comorbidities (cardiovascular, respiratory, or renal) and patients in whom massive intraoperative loss was expected (placenta accreta or placenta previa) were excluded.
Lal 2023 [22]	The study did not include patients with hypertension, increased intraabdominal pressure, body mass index (BMI) greater than 30 kg/m2, undergoing unilateral SA, and pregnant women. If, after recruiting, the level of anaesthesia achieved was above T5, then that patient was excluded from the analysis.
Moschovaki 2023 [28]	Exclusion criteria included patients with tachycardia (heart rate > 100 beats/min), atrial fibrillation or left bundle branch block on ECG. Poor acoustic windows, pulmonary hypertension (peak tricuspid velocity > 3.4 m/sec), tricuspid/ mitral/pulmonary valve regurgitation grade 3 or 4, severe aortic/mitral valve stenosis, or severe mitral annulus calci-fication on the preoperative echocardiogram (in the operating theater). Additionally, patients who had the maximum cephalad dermatomal extension of the spinal sensory block below T12 or arterial hypotension related to overt intraoperative bleeding (> 150 cc) were also excluded.
Ni 2022 [23]	Exclusion criteria were pre-existing hypotension (defined as systolic arterial pressure <90 mmHg or mean arterial pressure <60mm Hg), severe cardiovascular disease [unstable angina or ejection fraction <40%, implanted pacemaker/cardioverter, decompensated heart failure, and elevated pulmonary arterial pressure >40 mmHg (13, 17)], contraindication for spinal anesthesia, canal stenosis, pregnant patients, body mass index (BMI) >30 kg/m2, or failure to perform spinal anesthesia.
Salama 2019 [18]	Exclusion criteria were BMI more than 30 kgm^2, taking angiotensin converting enzyme inhibitors, pregnant women, emergency cases, absolute or relative contraindications to spinal anaesthesia, patients scheduled for unilateral spinal anaesthesia and failure to perform spinal anaesthesia. Also, patients with a baseline arterial SBP less than 90mmHg or mean arterial blood pressure (MBP) less than 70mmHg were excluded.

Saranteas 2019 [29]	Exclusion criteria were the following: patients with atrial fibrillation, right ventricle dysfunction, LV dyssynchrony of any etiology, significant mitral annular calcification (dense continuous calcification extending past the commissures into anterior annulus or calcification _270 degrees of total annular circumference),8 and severe valvular disease.
Sethi 2023 [30]	Patients with obesity (BMI >30kgm2), bleeding diathesis, on anticoagulants or angiotensin-converting enzyme inhibitors, and known allergy to local anesthetics were excluded.
Shifa 2020 [31]	Exclusion criteria were the following: hypertension or cardiovascular disease
Singh 2019 [25]	Patients who refused, posted for emergency lower segment cesarean section, had associated cardiovascular, respiratory, renal diseases or who received preloading of intravenous fluid were excluded from this study.
You 2022 [32]	The exclusion criteria were: hypertension during pregnancy; cardiovascular or cerebrovascular diseases; psychiatric diseases; contraindication of combined spinal-epidural anesthesia (CSEA); unwillingness to participate in this study.
Xu 2021 [33]	Exclusion criteria were the following: hypertension or cardiovascular disease
